# The relationship between health literacy, food literacy, and dietary choices—a systematic review

**DOI:** 10.3389/fpubh.2026.1769299

**Published:** 2026-04-08

**Authors:** Eva-Maria Steinke, Birgitta Hucke, Melanie Rohmann, Florian Tietz, Jan-Christoph Lewejohann, Matthias Nuernberger

**Affiliations:** 1 Department of Emergency Medicine, Jena University Hospital, Friedrich Schiller University, Jena, Germany; 2 Department of Pediatrics, Jena University Hospital, Friedrich Schiller University, Jena, Germany

**Keywords:** dietary behavior, food literacy, health literacy, health outcomes, nutrition literacy

## Abstract

**Objectives:**

Health-promoting behaviors play a key role in maintaining well-being. Health literacy (HL), food literacy (FL), and nutritional literacy (NL) encompass the knowledge, skills, and attitudes needed to make informed health and dietary choices, influencing outcomes beyond direct medical interventions. However, the interrelationships among HL, FL, and NL remain unclear. This systematic review aimed to identify and synthesize the current evidence on whether HL and FL (including NL as a related construct) are associated and whether one can predict the other. Studies were included only if they assessed at least two of these literacies.

**Methods:**

The review was conducted following the Preferred Reporting Items for Systematic Reviews and Meta-Analyses 2020 (PRISMA 2020) protocol, using eligibility criteria derived from the Population, Interest, and Context (PICo) framework (Population: adults; Interest: HL, FL, and NL; Context: health outcomes and dietary behavior). Searches were performed in MEDLINE (PubMed) and Scopus (Elsevier) using controlled vocabulary and free-text terms covering the key concepts of adults, HL, FL, and NL, as well as health or dietary outcomes. Study quality and potential bias were assessed using the Academy of Nutrition and Dietetics Quality Criteria Checklist (QCC).

**Results:**

The search yielded 1,491 records; after removing 194 duplicates, 1,297 studies were screened by title, abstract, and keywords. Following stepwise exclusions for irrelevance, missing data, or methodological shortcomings, 17 studies met all inclusion criteria. Across these studies, HL, FL, and NL were consistently associated with dietary behavior, nutritional knowledge, and food-related decision-making. However, the multidimensional nature of FL and NL was rarely captured comprehensively, as most studies focused on specific components rather than the whole constructs. Although some evidence suggested mutual influence between HL and FL/NL, the heterogeneity of measurement tools limited conclusions about the direction of prediction.

**Discussion and conclusion:**

The findings indicate that HL, FL, and NL are interrelated and together shape dietary behavior. Literacy-sensitive and context-aware interventions that address both individual capacities and structural factors can improve diet quality and health outcomes, depending on baseline literacy and socioeconomic context. While FL and NL share core competencies with HL, the evidence supports treating them as distinct constructs. Harmonization of measurement instruments and conceptual frameworks is needed to clarify their respective roles in predicting health outcomes in health promotion.

**Systematic review registration:**

https://www.crd.york.ac.uk/PROSPERO/view/CRD420250652344, identifier PROSPERO (CRD420250652344).

## 1 Background

### 1.1 You are what you eat!

The prevalence of obesity is increasing in the United States and Europe, with around 5 in 10 adults currently overweight (1, 2). At the same time, dealing with health-related information, acquiring knowledge about and motivation toward a healthy behavior and nutrition remains alarmingly low: one in every three to almost one in every two Europeans may not be able to comprehend health-related information (3). A similar number of participants in the European Food Information Council (EUFIC) 2023–2024 FL Survey misinterpreted nutrition information (4). In Germany, 6 out of 10 adults have limited health literacy (HL) and food literacy (FL) (5). The overweight and obesity of schoolchildren is also increasing, while their overall health is declining (6)—one out of five children or adolescents is overweight (7). The identified risk factors were a mix of inherent, behavioral, dietary, environmental, and sociocultural influences (7). However, the link between food and health is constantly debated. While it is obvious that healthy eating promotes health, the exact connection between knowledge about health and food is unknown (8).

### 1.2 Health literacy

The concept of HL originated in the United States during the 1970s, emerging alongside efforts to integrate health education into school curricula (9). Initial research in the United States and Canada in the 1980s defined HL as “using printed and written information to function in society” and an individual’s capacity to apply reading and numeracy skills within healthcare contexts (10). Numerous definitions of HL have since evolved. In summary, HL is the ability to obtain, process, and understand health information to make appropriate health decisions (11, 12). It encompasses skills such as interpreting health data, critical thinking, and navigating healthcare systems (11). Low HL is associated with poorer health outcomes, including increased hospitalizations and decreased use of preventive services (11). Nutbeam’s definition of HL, later adopted by the World Health Organization (WHO), describes it as “the personal, cognitive and social skills which determine the ability of individuals to gain access to understand and use information to promote and maintain good health” (13). Nutbeam identified three levels of literacy: functional (basic) literacy, interactive (communicative) literacy, and critical literacy (12), and he expanded the concept to include knowledge, motivations, empowerment, and self-reliance for informed health-related decision-making, highlighting its practical relevance to everyday life (11, 12). He also advocated for educating people on healthy eating and about “basic food groups for developing practical skills in food preparation and healthier food choices through supply-side” policy (12). In 2012, the WHO, through the European HL Consortium, incorporated the definitions by Nutbeams (12) and Sørensen et al. (11) into the three domains of health: managing illness (health care), risk reduction (disease prevention), and health maintenance (health promotion) (11, 14). The European Health Literacy Consortium also developed a 47-item tool to assess HL, based on an analysis of peer-reviewed definitions and conceptual frameworks (15), along with a shorter 16-item version (European Health Literacy Survey Questionnaire–16 items [HLS-EU Q16]) (16). The comprehensive version even included a question about understanding food packaging information (14). Following this, the European Health Literacy Survey (HLS-EU) was conducted between 2009 and 2012 among 1,000 individuals across eight European countries (14), finding that nearly half of the participants (47.4%) had limited HL, with the socioeconomic background exerting a strong influence (15). The Measuring Population and Organizational Health Literacy (M-POHL) Health Literacy Survey, conducted between 2019 and 2021, aimed to standardize an internationally comparable survey according to HL in 17 European countries, with methodological extensions and new topic literacies, such as digital, navigational, communicative, and vaccination HL (16–18). Across all participating countries, 13% of the participants scored an inadequate, 33% a problematic, and 40% a sufficient, and 15% an excellent HL level (17). The level values varied greatly across the countries, confirming the need for a contextual concept for each country (17). Liu et al. (19) offered an updated holistic definition centered on the ability to obtain, understand, and translate health information to maintain or improve health, highlighting diverse individual and social contexts.

### 1.3 Food and nutrition literacy

FL refers to the knowledge, skills, and behaviors needed to plan, select, prepare, and consume foods to meet nutritional needs (20). It includes understanding food systems, food preparation skills, such as shopping and preparing food, applied decision-making of informed food choices, and critical reflection on food’s implications for personal and social health (20), as well as environmental sustainability and economic benefits (21). Its concept is seen as most relevant for action-oriented health promotion (22). NL focuses specifically on the ability to understand and apply nutritional information (23), especially related to dietary behavior and food choices (21). It involves skills like reading food labels, understanding serving sizes, and evaluating nutrition information (23). While FL has a broader scope encompassing the entire food system and cultural aspects, NL concentrates on the nutritional aspects of food and their impact on health (22). Both concepts are crucial for promoting healthy eating habits and improving overall health outcomes (22, 24). In the absence of these skills, individuals are less likely to achieve nutritional awareness or make healthy eating choices (22). However, both literacies overlap in large areas. Both literacies are increasingly applied in health promotion interventions, highlighting their importance in shaping health behavior. In a systematic review, Krause et al. (22) identified six definitions of NL and 13 definitions of FL, concluding that both NL and FL are specific forms of HL, a view already articulated by Blitstein et al. in 2006 regarding NL (25). HL is the broader umbrella concept from which NL and FL have emerged (22). NL as a subset of FL, which itself is characterized by a broader, more applied, and behavioral comprehension of people’s engagement with food (22).

### 1.4 The link between HL and FL

It is reasonable to assume that HL and FL, as well as NL, are interrelated. However, the research landscape here is heterogeneous. This systematic review, therefore, aims to investigate the current literature to determine whether there is a connection between HL and FL. We have combined FL and NL for this purpose. Our key questions were as follows: Is there a connection between HL and FL? Can FL predict HL and vice versa?

## 2 Methods

### 2.1 Search procedure

The literature search was carried out in the MEDLINE and Scopus databases on 18 August 2025. The strategy combined controlled vocabulary terms (e.g., Medical Subject Headings [MeSH]) and free-text keywords related to three core concepts: (1) adults > 17 years old, (2) health literacy or food literacy, and (3) health outcomes or dietary behaviors. Search terms included variations such as “health literacy,” “food literacy,” “nutrition literacy,” “diet quality,” “dietary behavior,” and associated measurement instruments. The following search string in Title/Abstract was used:

(“adult*” OR “young adult*” OR “older adult*” OR “population*” OR “community” OR “public”)

AND

(“measure” AND “health literacy” OR “measure” AND “food literacy” OR “measure” AND “nutrition literacy” OR “measure” AND “dietary literacy” OR “nutrition knowledge” OR “food knowledge” OR “label comprehension” OR “nutrition label*” OR “food skills” OR “dietary skills”)

AND

(“diet quality” OR “dietary behavior*” OR “dietary intake” OR “food choice*” OR “nutrition behavior*” OR “eating behavior*” OR “fruit and vegetable intake” OR “sugar-sweetened beverage*” OR “weight gain” OR “weight status” OR “BMI” OR “metabolic health” OR “chronic disease risk” OR “health outcome*”).

**Figure 1:**
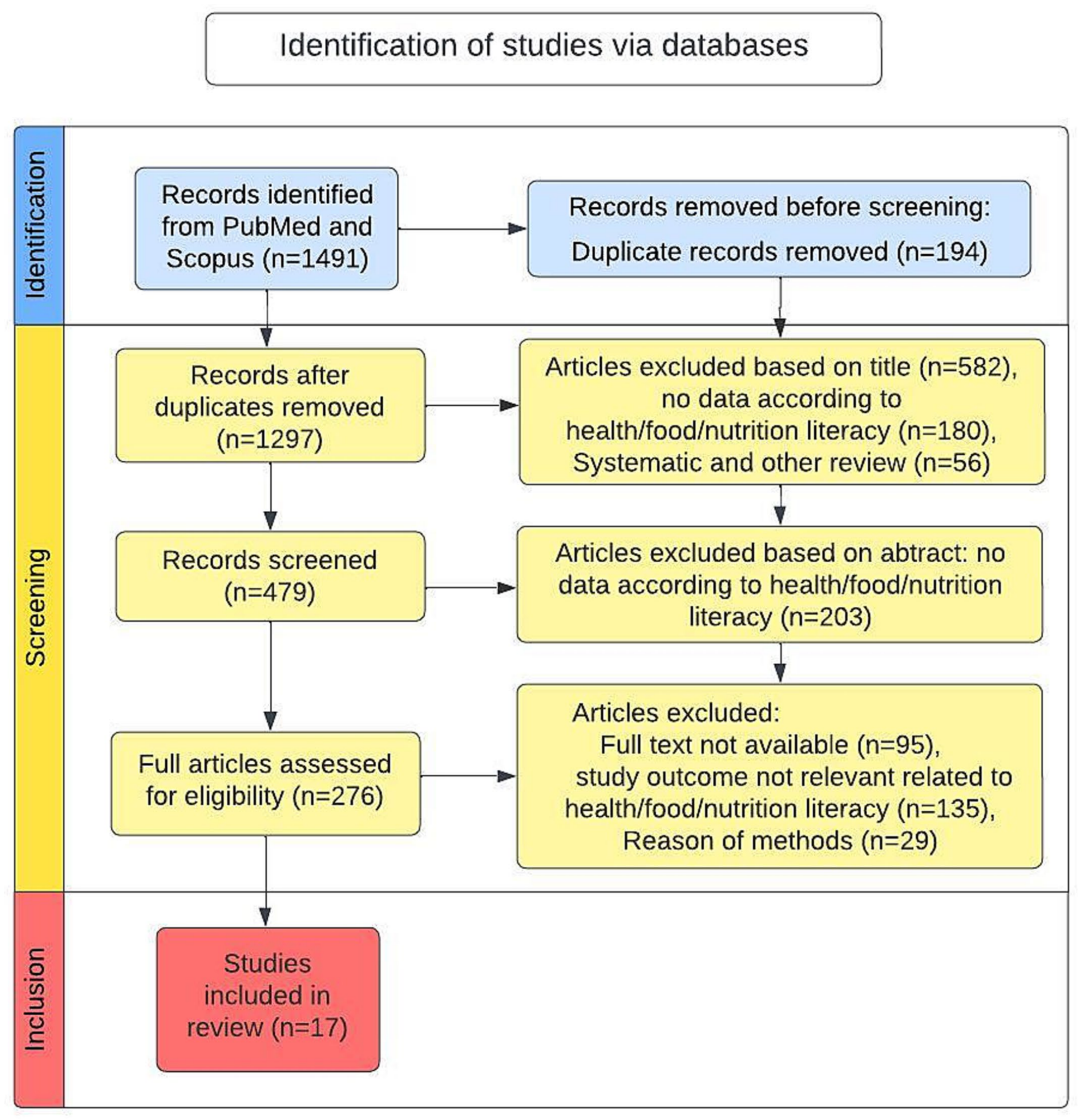
PRISMA 2020 search procedure flowchart (identification, screening, and inclusion).

The search strategy was adapted for each database. Language was limited to English. The search procedure is summarized in Figure 1. Both researchers reviewed the search strategy and the search terms. The researchers independently screened the articles for eligibility according to the criteria to reduce selection bias. The inclusion and exclusion criteria were set prior to the search.

### 2.2 Selection process

A systematic literature search was conducted in alignment with the Preferred Reporting Items for Systematic Reviews and Meta-Analyses 2020 (PRISMA 2020) (26) checklist (see Appendix 1). In accordance with PRISMA guidelines, two reviewers (MN and EMS) independently screened titles and abstracts against the predefined eligibility criteria. Studies that appeared relevant or for which eligibility was unclear were carried forward for full-text review. The same reviewers independently assessed the full texts to determine final eligibility. The initial criteria were the measurement of HL and FL. If neither had been assessed, the article was excluded. The data extraction included information about study design, year of publication, origin, and methods. At this stage, articles with the following criteria were excluded:

(1) The articles that did not measure health literacy and food literacy;
(2) The articles that included the wrong population, such as studies with children and adolescents, were excluded;
(3) Due to the wrong context of the articles;
(4) The articles that lacked relevant outcomes or empirical data; or.
(5) The inappropriate study type of the articles, such as qualitative studies.

The agreement rate of the studies included by both investigators was 92%. Disagreements at any stage were resolved through discussion. The final number of included studies was 17, as illustrated in the PRISMA flow diagram (Figure 1).

### 2.3 Eligibility criteria

Eligibility criteria were based on the standard PICo scheme (Population: adults > 17 years old; Interest: measure HL and FL; Context: health outcomes and dietary behavior). Studies with the following criteria were included the following:

- Language: English
- Study design: Randomized controlled trials (RCTs), non-randomized trials, cohort studies, case–control studies, cross-sectional studies, longitudinal studies
- quantitative design
- Topic: Provided information on HL and FL relevant to the key questions: Is there a connection between health and FL? Can FL predict HL and vice versa?
- Measurement of HL by a validated instrument
- Measurement of FL by a validated instrument

Studies with the following criteria were excluded:

- Study design: Systematic reviews or other types of reviews, conceptual articles;
- No access to full text;
- Employed incompatible and/or incomprehensible methods; or
- The article was not peer-reviewed.

### 2.4 Quality assessment

In accordance with the PRISMA checklist, the Academy of Nutrition and Dietetics (27) was used for quality assessment (see Appendix 2) due to its embedding in evidence-based practices and was developed by professionals with extensive expertise in nutrition science, food systems, and public health. This tool comprises 10 criteria to evaluate each study’s suitability for practical and scientific validity and to classify it as positive, neutral, or negative overall quality. For example, criteria are clear research questions, a referral to an intervention protocol and data collection process, an appropriate statistical analysis, and if results are supported by the conclusions. Quality assessment was performed by EMS and NM.

## 3 Results

All studies were digitally stored using Zotero version 1.0.3 and summarized in an Excel document as a PRISMA-compliant extraction form, as summarized in Table 1 as title, author, year, study design, study population, and measurement methods (see Table 1). 1,491 studies were found using the combined keyword-phrases, see Figure 1. After meticulous removal of duplicates (*n* = 194), we screened the remaining 1,297 studies by title, abstract, and keywords. A total of 56 studies were excluded because of being reviews. A total of 582 studies were excluded based on the title, and 180 records were removed because they revealed no data on HL or FL in the title. A total of 203 studies were excluded because they revealed no data on HL, FL, or NL in the abstract. A total of 276 articles were assessed for eligibility. A total of 95 studies were not available in full text; 29 studies did not meet methodological criteria; and the topics of 135 studies were not relevant to health, FL, or their connection. Respectively, 17 studies met the inclusion criteria and were included. These studies addressed HL, FL, or NL. Due to the complexity of the FL concepts, the studies focus on FL-related outcomes and the full FL concept is hardly pictured in the studies.

**Table 1:** Publications in the context of HL and FL.

Title	Author	Year	Study design	n	Age (mean, in years)	HL measured	FL measured
Health Literacy, Numeracy, and Health Promotion: A Secondary Analysis of the ChooseWell 365 Workplace Trial	Jia et al.	2022	RCT	602	43.5 ± 12.3	NVS, General Numeracy Scale (GNS, 3 items)	HEI-2015, Healthy Purchasing Score (HPS)
Randomized trial of planning tools to reduce unhealthy snacking: Implications for health literacy	Ayre et al.	2019	Pilot-RCT	440	46.2 ± 13.9	NVS	Seven-item snack score
Effects of a behavioral and Health Literacy intervention to reduce sugar-sweetened beverages: a randomized-controlled trial	Zoellner et al.	2016	Effectiveness-implementation hybrid RCT	296	42.1 ± 13.4	NVS, numeracy, REALM, Media Literacy	Beverage Intake Questionnaire (BEVQ-15), dietary recalls (3 non-consecutive days)
Nutrition Literacy among Cancer Survivors: Feasibility Results from the Healthy Eating and Living Against Breast Cancer (HEAL-BCa) Study: a Pilot Randomized Controlled Trial	Parekh et al.	2018	Pilot-RCT	59	58 ± 10.3	NVS	NLit-BCa, FVI, height, weight, BMI
Buenos habitos alimenticios para una buena salud: evaluation of a nutrition education program to improve heart health and brain health in Latinas.	Otilingam et al.	2015	RCT	100	59 ± 9	NVS	U. S. Department of Agriculture’s Diet and Health Knowledge Survey (nine questions) & Fat-Related Diet Habits Questionnaire
A randomized trial of a brief multimedia intervention to improve comprehension of food labels	Jay et al.	2009	RCT	61	51.5 ± 15	STOFHLA	Nutrition food-label quiz assessing food knowledge (not validated)
Role of Health Literacy profiles in fluid management of individuals receiving hemodialysis: A cross-sectional study	Chen et al.	2024	Cross-sectional study	433	55 ± 15	HLQ, Fluid Self-efficacy Scale, Fluid Adherence Subscale	Self-developed questions about food habits, Fluid Self-efficacy Subscale, Fluid Adherence Subscale
Exploring the Impact of Sociodemographic Characteristics and Health Literacy on Adherence to Dietary Recommendations and Food Literacy	Forray et al.	2023	Cross-sectional study	1,572	53 ± 17	HLS-EU-Q16	SFLQ, adapted FFQ
The Relationships between Food Literacy, Health Promotion Literacy, and Healthy Eating habits among young adults in South Korea	Lee et al.	2022	Cross-sectional study	325	18–33	Subscales of the European HL Survey Questionnaire (35)	SPFL (45), FL measurement scale (61)
Food and health promotion literacy among employees with a low and medium level of education in the Netherlands	Sponselee et al.	2021	Cross-sectional study	222	42.6 ± 13	health promotion literacy via 6 items of HLSEU-Q16	SPFL (45)
The Nutrition Literacy Assessment Instrument for Italian Subjects, NLit-IT: Exploring Validity and Reliability	Vettori et al.	2021	Cross-sectional observational study	74	38.8 ± 13.1	HL-EU-Q6 (Italian version)	NLit-IT, MEDI-Lite
Effectiveness of an education intervention associated with an exercise program in improving disease-related knowledge and health behaviors among diabetes patients	Ghisi et al.	2020	Quasi-experimental trial/ Intervention study	84	59.8 ± 11.4	NVS, Medical Term Recognition Test (METER), DiAbeTes Education Questionnaire (DATE-Q)	Self-administered version of the Mediterranean Diet Score (MDS) tool
Health Literacy and Nutrition Behaviors among Low-Income Adults	Speirs et al.	2012	Cross-sectional or survey-based study	142	37 ± 11	NVS	SFDHKS + nutrition behaviors (FVI, juice)
HL is associated with Healthy Eating Index Scores and Sugar-Sweetened Beverage Intake: Findings from the Rural Lower Mississippi Delta	Zoellner et al.	2011	Cross-sectional study	376	45 ± 16	NVS	Validated 158-item regional FFQ, HEI
Brief instruments for measuring nutrition literacy - the Nutrition Health Literacy Scale and the Self-Perceived Food Literacy Scale Short Form	Griebler et al.	2024	Validation study	2,993	48.3 ± 16.5	Nutrition HL Scale	Self-perceived FL Scale Short Form (SPFL-SF) (45)
Hungarian Adaptation and Validation of the Short Food Literacy Questionnaire (SFLQ-HU)	Keczeli et al.	2020	Validation study	1,325	27.7 ± 8.5	Brief Health Literacy Screening Tool (BRIEF)	SFLQ, Food Choice Questionnaire (FCQ)
A short Food Literacy Questionnaire (SFLQ) for adults: Findings from a Swiss validation study	Krause et al.	2018	Validation study	408	16–65, *Median* 43	HLS-EU-Q16	SFLQ

The 17 included studies were published between 2009 and 2024 and originated predominantly from high-income countries. Most studies employed cross-sectional designs (*n* = 7), followed by RCTs (*n* = 6), validation (*n* = 3), and intervention studies (*n* = 1). HL was measured using Newest Vital Sign (NVS; *n* = 8), HLS-EU-Q16 or subscales of it (*n* = 6), or different tools (*n* = 3). The instruments assessing FL broadly varied, including the Short Food Literacy Questionnaire (SFLQ; *n* = 3), Self-Perceived Food Literacy Scale (SPFL; *n* = 3), Healthy Eating Index (HEI; *n* = 2), Food Frequency Questionnaire (FFQ; *n* = 2), and seven different tools were used (see Table 1). Apart from Jay et al. (28), the five authors used the NVS for HL assessment, with 37% of Jia et al.’s (29) participants, 48.2% of Ayre et al.’s (30) participants, and 33% of Zoellner et al.’s (31) participants scoring low on HL. Parekh et al. (32) only provided a comparison of NVS scores (control group: 4.8 vs. intervention group: 5.0). In their study, Otilingam et al. (33) did not report NVS scores. Jay et al. (28) used the Short Test of Functional Health Literacy in Adults (STOFHLA) to measure HL and a nutrition knowledge and nutrition food-label quiz (pre- and post-intervention).

### 3.1 Association between HL and FL and food choices

The RCTs consistently demonstrated that HL is linked to FL-related outcomes, particularly how participants understood and benefited from nutrition or food-related interventions, and improved diet quality, nutrition label comprehension, or food purchasing behavior [Jia et al. (29), Ayre et al. (30), Zoellner et al. (31), Jay et al. (28), Otilingam et al. (33)]. Several studies showed that individuals with lower HL strongly profited from HL-sensitive interventions, as simplified language, visual cues, traffic-light labels, nudging strategies, and reduced cognitive load (29, 30). Diet quality (29) and snacking behavior (30) had been more effectively addressed. Accordingly, the effectiveness of the interventions depended on baseline HL. In contrast, Zoellner et al. (31) found no moderating effect of baseline HL on behavior-change outcomes, as participants with low and high HL showed comparable liability and reductions in sugar-sweetened beverage intake when exposed to a highly HL-sensitive intervention. With the exception of Body Mass Index (BMI), no significant between-group effects were found for other biological outcomes (e.g., blood lipids and glucose) within the 6-month timeframe. The study shows that an HL-sensitive intervention (simplified materials, teach-back calls, culturally appropriate delivery) influences FL-related behavior by reducing sugar-sweetened beverage (SSB) intake. Ayre et al.’s (30) Australian participants did not differ significantly in their reduction of snacking across the three arms when ignoring HL. The significant interactions between the intervention effects and HL were also reported by Jia et al. (29). Both studies indicate a dependence of baseline HL on improvements in food-related behavior and in healthy eating purchases. Jay et al. (28) demonstrated that HL was associated with FL-related competencies, especially the understanding of nutrition facts labels. The multimedia approach improved food label comprehension for people with adequate HL. Still, it was insufficient for those with limited HL, highlighting HL as a prerequisite for certain FL outcomes when interventions are not sufficiently tailored. The study by Parekh et al. (32) demonstrates that an education-based intervention to improve NL (understanding and use of nutrition-specific information) is feasible, even in a clinical population like cancer survivors. The authors of the RCTs Jia et al. (29), Ayres et al. (30), Zoellner et al. (31), and Jay et al. (28) reached a consistent conclusion. Interventions might need to combine education with skills-building or environmental support (e.g., easier access to healthy food, practical cooking guidance). There is a context-dependent connection between HL and FL, particularly recognizable when FL outcomes rely on comprehension, interpretation, and application of food-related information.

Within the cross-sectional studies, Forray et al. (34), Lee et al. (35), and Sponselee et al. (36) measured HL with subscales or the full HLS-EU-Q16, and FL with the SFLQ or SPFL. Ghisi et al. (37), Speirs et al. (38), and Zoellner et al. (31) relied on NVS for HL assessment. For FL, Ghisi et al. (37) applied the Mediterranean Diet Score (MDS) tool, Speirs et al. (38) drew on the Short Format of the Diet Health and Knowledge Survey (SFDHKS) and nutrition behaviors (fruit and vegetable intake; FVI, juice), and Zoellner et al. (31) employed a validated 158-item regional Food Frequency Questionnaire (regional FFQ) and the HEI.

The cross-sectional studies consistently reported positive associations between HL and FL-related behavior. Higher HL was associated with better self-reported FL skills, healthier dietary choices, and higher diet quality (35, 37–41). Speirs et al. (38) and Ghisi et al. (37) showed significant associations between HL and specific food practices (e.g., higher fresh fruit intake, less frying, healthier food choice). Ghisi et al. (37) and Chen et al. (41) concluded that a stronger HL (understanding, processing, and using health information) and self-efficacy boosted confidence in behavior change and healthier dietary choices. Speirs et al. (38) argued, as Jia et al. (29), Ayres et al. (30), Zoellner et al. (31), and Jay et al. (28), that health and nutrition information should be rendered more accessible to individuals with low HL, as they often face challenges in applying such information to everyday dietary decisions. While the study by Speirs et al. (38) does not explicitly mention FL, behaviors, such as food-label use, food preparation choices, and fruit consumption align with FL components. Chen et al. (41) suggest that interventions should be person-centered, considering patients’ HL level and cultural habits. Not all studies identified strong associations. Sponselee et al. (36) found a small positive correlation between FL and HL in adults. Similarly, Vettori et al. (40) found no significant correlation between HL and NL levels (as assessed by HLS-EU-Q6 scores and NLit-IT scores). Whereas mean HL scores were adequate, NL scores indicated the possibility of poor NL.

### 3.2 Association between HL and FL

Lower HL was linked to lower FL skills and poorer adherence to dietary recommendations (34). Forray et al. (34) reported that 42.9% of participants had sufficient HL, while 16.3% had excellent levels. Individuals with higher HL reported better FL, were able to identify healthy foods or seek nutrition information, and promoted adherence to dietary recommendations. Forray et al. (34) and Zoellner et al. (39) linked HL to objective indicators of diet quality, such as serum carotenoid levels and sugar-sweetened beverage intake. Gender (35) and self-efficacy (37) influenced the relationship between HL and FL. When female participants had higher FL scores, Lee et al. (35) found in their analysis that the relationship between FL and healthy eating habits was significant only among woman, implying that gender exerts a moderating effect.

The results across the validation studies by Griebler et al. (42), Krause et al. (43), and Keczeli et al. (44) showed that FL and nutritional HL (NHL) or HL were positively associated, supporting conceptual overlap. Griebler et al. (42) developed and validated an instrument to measure Nutrition HL Scale (NHLS) and a shortened version of the Self-Perceived FL Scale (SPFL-SF) by Poelman et al. (45) among 2,993 adults in Austria. The study aimed to create tools for a broader public health approach, with the SPFL-SF offering a more concise measure of behavioral and skills-based competencies, while the NHL focuses on nutrition information processing. Krause et al. (43) developed and validated a brief questionnaire (SFLQ, 12 items) and found a positive association with HL scores (i.e., people with higher HL tended to score higher on FL) and with nutrition knowledge among approximately 350 adults in Switzerland. While it is correlated with FL, the SFLQ focuses more specifically on food-related behaviors, knowledge, and skills, and investigates how improving FL might influence dietary behavior and health outcomes. The finding of a positive correlation between HL and FL suggests that interventions to raise HL may benefit FL (and vice versa). Alike, the study by Keczeli et al. (44) validated the SFLQ for the Hungarian population (SFLQ-HU), finding a positive correlation with HL constructs. Individuals with higher FL also tended to have better health information competence. The findings reinforce that HL and FL share common domains related to information processing, decision-making, and applied skills, while remaining analytically separable.

## 4 Evaluation of quality and strength of evidence

The Academy of Nutrition and Dietetics Quality Criteria Checklist (QCC) (27) was used for quality assessment (see Appendix 2). This checklist comprises 10 criteria to evaluate each study in terms of its suitability for practical and scientific validity, and to classify it as positive, neutral, or negative overall quality. The body of evidence varied in strength, ranging from low to moderately strong. All studies defined a clear research question, referred to an intervention protocol and data collection process, and ran appropriate statistical analysis. The conclusions supported the results. The studies showed considerable variation in participant age range, sample size, and methods, as well as the lack of validated literacy instruments (see Table 1). Every single study has not reported the funding of the study. This review examines the following six RCTs assessing the effectiveness of nutrition education and FL interventions: Jia et al. (29), Ayre et al. (30), Zoellner et al. (31), Parekh et al. (32), Otilingam et al. (33), and Jay et al. (28). These studies varied considerably in sample size, follow-up duration, population, intervention design, and outcome measures, resulting in heterogeneous evidence quality. Five of the six RCT studies [Jia et al. (29), Ayre et al. (30), Zoellner et al. (31), Parekh et al. (32), and Otilingam et al. (33)] demonstrated positive overall outcomes. Limiting confidence in robustness and generalizability is due to small sample sizes by Parekh et al. (32) (*n* = 59) and Jay et al. (*n* = 42), who used an unvalidated outcome measure. Follow-up periods ranged from 1 month (30) to 24 months (29), with (31) assessing participants at 6, 12, and 18 months. HL assessment varied as five studies employed the NVS, whereas Jay et al. (28) used the Short Test of Functional Health Literacy in Adults (STOFHLA). Only Ayre et al. (30) blinded participants to the intervention, which, combined with a reasonable sample size and a validated outcome, contributed to its moderate overall score, given the improvement in food-label comprehension among adults with adequate HL. For broader claims (behavior change, health impact), evidence is lacking. Outcome assessment tools varied widely; some measured general HL, others FL or NL, or specific domains, thereby complicating comparability. Validated dietary measures included Ayre et al.’s (30) seven-item snack score, Zoellner et al.’s (31) BEVQ-15 and dietary recalls, Parekh et al.’s (32) NLit-BCa and FVI, and Otilingam et al.’s (33) Health Knowledge Survey and Fat-Related Diet Habits Questionnaire. Jay et al. (28) used a non-validated nutrition food-label quiz. Across studies, reliance on self-reported dietary data introduced potential recall and social desirability biases. Jia et al. (29) demonstrated moderate-quality evidence, though generalizability is limited. Ayre et al.’s (20) results were partly supported by validated measures. Zoellner et al. (31) provided moderate to moderately strong evidence that an HL-oriented intervention supports causal inference for behavior change. Parekh et al. (32) offered weak-to-moderate evidence, while methodologically sound (randomization, validated instrument, good reporting, feasibility), the small sample, short follow-up, and limited outcomes restricted its impact. Otilingam et al. (33) demonstrated moderate evidence, but results were limited in scope and did not demonstrate long-term or objective health outcomes. Jay et al. (28) provided low-to-moderate evidence due to the intervention did not reliably influence dietary behavior, nutrient intake, or long-term outcomes. Overall, these RCTs highlight that brief, targeted interventions can enhance nutrition knowledge and literacy, particularly in participants with adequate HL. However, translation into sustained long-term behavioral changes, clinically relevant health outcomes, and applicability across broader or more diverse populations remains limited. The use of validated FL measurement tools in parallel with the intervention was rarely found.

Across the studies reviewed by Chen et al. (41), Forray et al. (34), Lee et al. (36), Vettori et al. (40), Ghisi et al. (37), Speirs et al. (38), Zoellner et al. (39), Griebler et al. (42), Keczeli et al. (44), and Krause et al. (43), there is consistent moderate to good-quality evidence indicating associations between HL and FL and diet-related behaviors in adults. Lee et al. (35) and Speirs et al. (36) provided suggestive but low evidence suggesting a possible link between HL and food-related behavior. Eight publications [Chen et al. (41), Forray et al. (34), Lee et al. (35), Sponselee et al. (36), Vettori et al. (40), Ghisi et al. (37), Speirs et al. (38), Zoellner et al. (39)] employed cross-sectional designs, limiting causal inference but demonstrating robust correlational patterns. These studies had large sample sizes, solid statistical analyses, and utilized a wide range of literacy assessment tools: Forray et al. (34), Lee et al. (35), and Sponselee et al. (36) applied subscales of the HLS-EU-Q16, Vettori et al. (31) employed the HLS-EU-Q6, and Ghisi et al. (37), Speirs et al. (38), and Zoellner et al. (39) used the NVS. Chen et al. was the only author to apply the HLQ, a comprehensive, 44-item, multidomain tool that captures both individual capacities and health-system responsiveness, thereby providing deeper insights than assessments based solely on functional reading skills.

FL and dietary behavior were also measured using varied tools across studies. Forray et al. (34) applied the SFLQ along with an adapted FFQ, while Zoellner et al. (39) used a regional FFQ combined with HEI scoring. Lee et al. (35) and Sponselee et al. (36) relied on the SPFL. Vettori et al. (40) and Ghisi et al. (37) focused on Mediterranean diet indices (Mediterranean Diet Literature-Based Adherence Score [MEDI-Lite] and versions of the Mediterranean Diet Score) and Vettori et al. (40) additionally incorporated the NL for Italians (NLit). Chen et al. (41) used self-developed items addressing food habits, fluid adherence, and self-efficacy, whereas Speirs et al. (38) applied the Short Food, Dairy, and Health Knowledge Scale (SFDHKS) alongside nutrition-behavior items.

The validation studies by Griebler et al. (42), Keczeli et al. (44), and Krause et al. (43) further examined HL and FL measurement tools. These studies demonstrated strong psychometric properties for instruments such as the NHLS, BRIEF HL screener, HLS-EU-Q16, SPFL-SF, and SFLQ, providing high-quality evidence for measurement validity. All studies showed consistent associations emerged between lower HL or FL and poorer diet quality, lower adherence to dietary guidelines, and less frequent engagement in health-promoting food practices. Across all evidence, several methodological limitations affect the strength and generalizability of findings. The exclusive recruitment of young university students in the studies by Lee et al. and Keczeli et al. (44) introduces selection bias and limits their applicability to broader populations. Vettori et al. (40), Griebler et al. (42), and Keczeli et al. (44) did not report participant response rates, reducing transparency and potentially inflating sampling bias. Funding bias may have influenced findings in studies by Ghisi et al. (37) and Zoellner et al. (39). Additionally, the reliance on self-reported literacy, dietary behavior, and perceived skills introduces social desirability bias, particularly in health- and food-related contexts, and many outcomes were not validated using objective external measures. However, because most studies were cross-sectional and dependent on self-reported data, the strength of evidence for causal relationships remains low. One intervention study (37) tested the impact of educational strategies, showing short-term improvements but did not isolate literacy-specific effects or demonstrate long-term outcomes. Nevertheless, the trial does not isolate the specific impact of literacy, is limited by sample size, and lacks long-term follow-up.

Overall, the evidence is strong for the validity of literacy measurement tools and moderate for describing literacy-diet associations, but weak for establishing that improving HL leads to advanced FL (or vice versa) or that literacy alone leads to sustained dietary or health improvements. Health outcomes (e.g., weight, metabolic disease risk) often require longer follow-up, and short-term changes may not reflect long-term benefits. The effectiveness might differ in urban populations, other countries, or more diverse socioeconomic and cultural contexts. The findings help identify vulnerable populations and highlight the need for longitudinal and experimental research to clarify causal pathways and long-term public-health impacts.

## 5 Discussion

### 5.1 Intervention evidence: behavior change and literacy-sensitive approaches

Intervention research provides more robust insight into whether literacy actively drives behavior change. Zoellner et al.’s (31) work is particularly important because it used an HL-tailored behavioral intervention within an RCT design, allowing relatively strong causal inference. The intervention explicitly accounted for participants’ varying HL, mirroring the distribution found in the general population and thereby improving real-world applicability. Literacy-sensitive behavioral strategies reduced SSB consumption and improvemed BMI, indicating that behavior change had meaningful effects on a health-relevant parameter. Although focused on a specific dietary behavior, it connects HL to food-related behavior change, linking HL and FL. The findings suggest that FL interventions should use a literacy-sensitive design (clear language, support, and repeated contact) to reach individuals across literacy levels. However, long-term health impacts cannot be inferred. Still, the combination of real-world delivery and an HL-tailored RCT demonstrates that literacy-sensitive public health interventions are both feasible and effective. As Glenton et al. (46) stated, is this an important methodological insight for addressing complex public-health problems shaped by behavior, context, and social factors?

A European review by Visscher et al. (47) similarly found that HL interventions, especially those that are multimodal, tailored, and culturally appropriate, can promote prevention behaviors and improve outcomes, particularly among disadvantaged populations. Interventions that use interpersonal support or skills-based activities outperform those rely solely on information delivery. Otilingam et al.’s (33) culturally adapted education program demonstrated improvements in HL, nutrition knowledge, and fat-reduction behaviors, reinforcing the value of integrated, culturally sensitive approaches. Nevertheless, gaps persist; very few high-quality HL interventions target older adults. Theory-based programs are generally effective; however, substantial variation in design and outcomes limits generalizable conclusions.

Tailored interventions have the potential positively affect literacy. In the workplace-based dietary intervention by Jia et al. (29), individuals in the intervention group with lower HL responded more favorably to food-labeling and environmental changes than those in the control group. A meta-analysis found that labeling can modestly reduce energy and fat intake and prompt industry reformulation (48). Evidence also shows that label education alone often produces modest, context-dependent changes. An RCT testing traffic-light labels led to better identification of healthy foods, but only small or no changes in actual choices (49). A systematic review of 17 studies found that while label-education programs improve comprehension, most did not track real dietary behavior or health outcomes and had short or absent follow-up (50). More recent studies confirm modest, unsustained effects of label-based interventions, although multimedia formats can improve comprehension more effectively than text for some groups (51).

Additional evidence highlights the role of baseline HL in shaping the effectiveness of interventions. Ayre et al. (30) further demonstrated that a brief multimedia HL intervention improved short-term label comprehension, but only among adults with adequate baseline HL. Jay et al. (28) similarly found the greatest gains in individuals with adequate HL in the intervention group, with no improvement in participants with limited HL. This restricts generalizability; the people who could benefit most, those with low HL, may be least able to use such interventions. Consequently, one-size-fits-all approaches are insufficient. Tailored strategies or additional support are needed to ensure equity in health behavior change. Accordingly, both studies return to and reinforce the findings of Jia et al. (29) and Zoellner et al. (31) that intervention design must depend on HL. The study by Zoellner et al. (31) provided actionable evidence that behavioral, literacy-tailored interventions can reduce sugar-sweetened beverage consumption and modestly affect BMI in adults. Accessible and simplified interventions, as well as interactive materials, may have a more effective impact on the nutritional quality of people with varying levels of HL and knowledge. These findings suggest a functional link between HL and FL, in which HL moderates the translation of nutritional information into food-related behavior.

Other FL-focused interventions show modest but positive short-term effects. Begley (52) reported small improvements in planning, selection, preparation, and general dietary behavior following FL programs. Parekh et al. (32) evaluated a multimodal nutrition-education intervention (lectures, interactive components, and cooking sessions) among breast-cancer survivors, finding short-term improvements in NL, knowledge, and FVI. However, small sample sizes and mixed results limit the ability to draw strong conclusions. Still, the study contributes to the emerging evidence that HL interventions can support self-management in chronic disease populations (53).

### 5.2 Measurement tools and methodological advances

Advances in measurement are helping resolve long-standing challenges. Vettori et al.’s (40) validation of the NLit-IT shows that NL is a distinct construct, moderately associated with diet quality but separate from general HL (54). Thus, the study contributes to the evidence base showing that improving NL could be one contributing factor in public health to support healthier diets, such as the Mediterranean Diet, and therefore complements efforts to raise general HL. Validated instruments are essential for reproducibility, allowing cross-cultural comparisons and more rigorous testing of literacy-diet-health models.

Griebler et al. (42) and Krause et al. (43) emphasized that the field suffered for years from heterogeneous, unvalidated instruments, making cross-study synthesis challenging. Newer tools, such as SFLQ, SPFL, and NLit-IT, offer improved reliability. However, some reduce literacy to a single, context-specific dimension, potentially overlooking important components such as cooking skills, planning, budgeting, or critical evaluation. In general, it underscores that the conceptualization of FL is measurable, useful for detecting FL gaps across the population, identifying vulnerable groups, monitoring over time, evaluating programs, and fostering public health and policy work. Validation studies suggest that higher HL is associated with stronger health information competence, implying potential bidirectional reinforcement.

### 5.3 HL and NL as predictors of behavior

Across diverse populations, HL, FL, and NL consistently relate to dietary behavior and self-management, though cross-sectional designs often limit causality. Chen et al. (41) found that higher HL was associated with lower relative interdialytic weight gain. The findings among hemodialysis patients align with those of Lim et al. (55), who found that FL independently predicted dietary adherence and mediated the relationship between diet, self-efficacy, and self-management skills (55). A broader review shows that limited HL is common in patients with chronic kidney disease and associated with poorer adherence, more hospitalizations, and higher mortality (56).

In the general population, Forray et al. (34) demonstrated that Romanian adults with lower HL or FL were less likely to meet dietary recommendations (fruit/vegetables, fish, water). Diet adherence is influenced not only by literacy but also by structural and social factors. Lower literacy is clustered with structural disadvantages (older age, rural residence, and lower education), suggesting that literacy deficits mirror systemic inequalities. The findings suggest that enhancing HL may improve FL, leading to better dietary adherence. Individuals with advanced HL also tend to feel more confident in food-related decisions. This supports the view that FL is integrated within broader HL, with effective management of health behaviors, including diet, that rely on both domain-specific FL skills and general health literacy. Still, because the study was cross-sectional and self-reported, it cannot show that literacy improvement would automatically lead to healthier diets or overcome barriers such as affordability or access.

Similar associations appear in young adults. Lee et al. (35) found that all dimensions of FL (knowledge, skills, resilience) and HPL were positively associated with healthy eating, with gender moderating some of these relationships. However, here too, the cross-sectional design only justifies the claim that higher HL may be associated with healthier eating habits. Accordingly, the results support the conclusion that FL is linked to but distinct from HL, and both shape dietary behavior. Limited HL often constrained improvements in FL unless interventions were highly tailored or supported. HL appears to be a key enabling factor for FL, particularly for cognitively demanding tasks such as interpreting nutrition labels and applying dietary guidelines. It further emphasizes that interventions targeting diet or food practices should incorporate HL considerations (e.g., comprehension skills and critical evaluation of health/nutrition information) rather than addressing FL in isolation. An inextricable linkage between food and health has also been confirmed by prior findings by Vidgen and Gallegos (20), and Poelman et al. (45). Japanese studies reinforce this, as individuals with stronger FL (cooking skills, budgeting, meal planning, and label reading) showed higher HEI scores (57). These findings collectively suggest that literacy supports healthier choices, even though directionality and causality remain uncertain.

Integrating HL education into FL programs can enhance both knowledge and the confidence to act. Broad dietary changes and HL improvement likely require complementary strategies, including simplified language, visual aids, and teach-back methods to ensure understanding. However, this construct also offers explanations that improving one literacy does not improve the other in the same way. Therefore, health promotion programs should prioritize enhancing both FL and HL, rather than focusing on either domain in isolation. Literacy helps, but it is no barrier against systemic disadvantage. Evidence from Taylor (58) and Zoellner et al. (39) shows that even when literacy predicts healthier dietary patterns, external factors strongly determine whether people can act on their knowledge. The findings by Keczeli et al. (44) and Krause et al. (43) support the idea that FL is linked to HL, yet distinct enough to require separate measurements. FL, specific to food-related behavior, can be considered as a subdomain of HL, but evidence for FL as an independent predictor of general HL is limited and indirect. The study highlights the importance of independently measuring FL better to understand food choice behaviors in nutrition education and interventions. These findings underscore the value of assessing both HL and FL in research and practice, as each provides unique insight into factors shaping food choices and nutrition-related health outcomes.

### 5.4 Remaining uncertainties and evidence gaps

Despite progress, several uncertainties persist. There is a conceptual ambiguity because definitions of FL or NL vary widely, complicating comparisons (43). Measurement often is heterogeneous. Most interventions improve knowledge or short-term behavior but lack long-term dietary or health outcomes (50, 59). Intervention effectiveness often varies by baseline HL and socioeconomic status, yet many studies fail to analyze or report this heterogeneity (60). There are structural influences, as literacy alone cannot overcome barriers such as affordability, environment, or cultural norms.

### 5.5 Integrative perspective

Taken together, the evidence portrays literacy as a meaningful but insufficient determinant of healthy behavior. HL and FL consistently relate to dietary patterns, and tailored, multimodal interventions, especially those sensitive to baseline literacy, can produce modest but real improvements in behavior and intermediate health indicators. RCTs such as Zoellner et al.’s (31) demonstrate that literacy-sensitive behavior change is feasible and impactful. Advances in validated measurement tools strengthen the research foundation. The comparable use of both HL and FL with validated and reliable FL measurement tools remains the major challenge. Still, longitudinal or intervention studies are needed to evaluate the predictive value of HL and FL. FL may enhance nutrition-specific behavior, but evidence is weak. HL and FL should be viewed as complementary constructs, addressed collaboratively through HL-sensitive, skill-based, and context-aware interventions.

### 5.6 Limitations of this review

This study is subject to limitations related to keyword definitions and language restrictions, as only articles in English language were included, reducing generalizability of the results, the search methodology, which excluded publications not available in full text. Further, the authors subjectively assessed whether the study methods were reliable and whether the data or study outcomes were relevant to HL, FL, and NL. No librarian was involved in the search process. A relevant part of the included studies had a cross-sectional design (34–36, 39–41), which does not allow causal inference. Contextual and population limitations have to be acknowledged. Most studies were conducted in high-income countries and specific demographic groups, which limits generalizability to other populations, particularly those with different cultural, socioeconomic, or food-system contexts. The review gives a comprehensive coverage of HL and FL measurement approaches. The variability in parameters and methods, especially with adapted questionnaires (19, 20, 28, 34, 41), poses another limitation by reducing the possibility of standardized comparison. The lack of a standardized and uniform way to assess FL is also a relevant limitation. Measurement tool to validate FL predominantly emphasizes numeracy skills and literacy or nutrition knowledge. The often-used NVS test concentrates on individuals’ ability to effectively read food labels. Other instruments focus on broader skills and characteristics of nutrition knowledge. Distal outcomes such as dietary intake, anthropometric measures, or general health indicators without assessing core literacy components, including food-related knowledge, skills, critical understanding, or decision-making capacity, limit the ability to draw firm conclusions about the role of literacy itself. This variability reduces comparability across studies and may have contributed to inconsistent findings. The multidimensional nature of HL and FL was difficult to capture within the study designs and limited the robustness of the evidence.

### 5.7 Areas for further research

Future research should aim to standardize measurement tools to assess different literacies. Surveys should continuously screen the actual status of various literacies across populations and generate measures to update results from the latest research and their transfer into application practice. Especially in times of Internet and an increasingly complex food system, the necessity is given to prepare individuals with knowledge, exercises, and strategies to be able to make informed health decisions for improving or protecting their quality of life.

## 6 Conclusion

FL is conceived as a subarea of HL due to complementary characteristics of core basic competencies, but must be regarded as an independent construct from HL that justifies the need for a separate measurement instrument. The current evidence portrays HL and FL as promising but not standalone targets for improving diet and health. In summary, evidence is strong for the validity of literacy measurement tools and moderate for literacy-diet associations, but remains insufficient to demonstrate that improvements in HL translate into higher FL (or vice versa), or that literacy independently drives long-term dietary or health outcomes. Higher levels of HL and FL, coupled with educational and socioeconomic background, also foster healthier behaviors and a more nutritious diet. While associations with healthier behaviors are robust, causality and long-term impact remain uncertain. Future research should integrate validated and tailored tools, methods, longitudinal designs, and literacy-level-appropriate intervention studies that consider age groups, ethnicities, socioeconomic status, and environmental determinants to understand better the role of literacy in promoting sustainable, healthy dietary behaviors.

## Data Availability

The original contributions presented in the study are included in the article/Supplementary material, further inquiries can be directed to the corresponding author.

## Glossary

BEVQ-15: Beverage Intake Questionnaire
BMI: Body Mass Index
BRIEF: Brief Health Literacy Screening Tool
CFL: Critical food literacy
CFNL: Critical food and nutrition literacy
CHL: Critical health literacy
CNL: Critical nutrition literacy
DATE-Q: DiAbeTes Education Questionnaire
FCL: Food choice literacy
FFL: Food frequency list
FFNL: Functional food and nutrition literacy
FCQ: Food Choice Questionnaire
FFQ: Food Frequency Questionnaire
FHL: Functional health literacy
FL: Food literacy
FLAT: FL assessment tool
FLI: Fatty Liver Index
FLL: Food label literacy
FNLIT: Food and nutrition literacy
FNL: Functional nutrition literacy
FVI: Fruit and vegetable intake
GNS: General Numeracy Scale
HbA1c: Hemoglobin A1c
HBSC: Health Behavior in School-Aged Children Survey
HEI: Healthy Eating Index
HFI: Healthy Food Index
HKC: Healthy Kids Check
HL: Health literacy
HLQ: Health Literacy Questionnaire
HLS-EU-Q16: European Health Literacy Survey Questionnaire – 16 Items
HPL: Health promotion literacy
HPS: Healthy Purchasing Score
IFL: Interactive food literacy
IFNL: Interactive food and nutrition literacy
IHL: Interactive health literacy
INL: Interactive nutrition literacy
MEDI-Lite: Mediterranean Diet Literature-Based Adherence Score
MeSH: Medical Subject Headings
MDS: Mediterranean Diet Score
METER: Medical term recognition test
NL: Nutrition Literacy
NHL: Nutritional Health Literacy
NHLS: Nutrition Health Literacy Scale
NLit-BCa: Nutrition Literacy Assessment Instrument for Breast Cancer
Nlit-IT: Nutrition Literacy Assessment Instrument for Italian people
NVS: Newest vital sign
PRISMA: Preferred Reporting Items for Systematic Reviews and Meta-Analyses
QCC: Academy of Nutrition and Dietetics Quality Criteria Checklist
RCT: Randomized controlled trial
REALM: Rapid Estimate of Adult Literacy in Medicine
SFLQ: Short Food Literacy Questionnaire
SF: Short form
SFDHKS: Short Format of the Diet Health and Knowledge Survey
SPFL: Self-Perceived Food Literacy Scale
UFI: Unhealthy Food Index
WHO: World Health Organization
