# Supplementary material for: The relationship between health literacy, food literacy, and dietary choices—a systematic review
Source: Front Public Health. 2026 Apr 8;14:1769299. doi: 10.3389/fpubh.2026.1769299 (PMC13099290; doi:10.3389/fpubh.2026.1769299)
Supplement: Supplementary file 2 [file Table_2.DOCX]

| **Criteria Scores** | | | | | | | | | | | | |
| --- | --- | --- | --- | --- | --- | --- | --- | --- | --- | --- | --- | --- |
| **Study** | **1. Clear research question** | **2. Participant selection free from bias** | **3. Comparable study groups** | **4. Description of participant withdrawals or response rate** | **5. Use of blinding** | **6. Description of intervention protocol and/or data collection procedures** | **7. Outcomes clearly defined, valid and reliable**  **measurements** | **8. Appropriate statistical analysis** | **9. Conclusions supported by results** | **10. Unlikely funding bias** | **Overall quality rating** |  |
| Jia et al. | + | + | + | + | Not possible | + | + | + | + | + | + |  |
| Ayre et al. | + | + | + | + | + | + | + | + | + | + | + |  |
| Zoellner et al. (2016) | + | + | + | + | NR, rather - | + | + | + | + | + | + |  |
| Parekh et al. | + | * | + | + | - | + | + | + | + | + | + |  |
| Otilingam et al. | + | + | + | + | Ø | + | + | + | + | + | + |  |
| Jay et al. | + | * | + | + | - | + | Ø | + | + | + | Ø |  |
| Chen et al. | + | + | N/A | + | N/A | + | + | + | + | + | + |  |
| Forray et al. | + | + | N/A | + | N/A | + | + | + | + | + | + |  |
| Lee et al. | + | - | N/A | + | N/A | + | + | + | + | + | Ø |  |
| Sponselee et al. | + | + | N/A | + | N/A | + | + | + | + | + | + |  |
| Vettori et al. | + | * | N/A | NR | N/A | + | + | + | + | + | Ø |  |
| Ghisi et al. | + | * | N/A | + | N/A | + | + | + | + | Ø | +/Ø |  |
| Speirs et al. | + | + | N/A | + | N/A | + | + | + | + | + | + |  |
| Zoellner et al. (2011) | + | + | N/A | + | N/A | + | + | + | + | NR | + |  |
| Griebler et al. | + | + | N/A | NR | N/A | + | + | + | + | + | + |  |
| Krause et al. | + | + | N/A | + | N/A | + | + | + | + | + | + |  |
| Keczeli et al. | + | - | - | Ø | - | + | + | + | + | + | Ø |  |

**Appendix 2:** Quality assessment attributes for each study assessed using the Quality Criteria Checklist (QCC)

‘+’: positive overall score: is given if criteria 2, 3, 6 and 7 of the QCC and one additional criteria have received a positive score; ‘Ø’: neutral overall score: is given if more criteria are met than for a negative overall score but a positive overall score is not reached; N/A; not applicable, NR: not reported; ‘-‘: negative overall score: is given if six or more QCC criteria are not met; ‘*’: not free of participants bias due to small sample size, missing specification according inclusion and exclusion criteria and /or missing description of subjects characteristic.
